# Investigating sustainable management of desalination brine through concentration using forward osmosis

**DOI:** 10.1007/s11356-021-13311-z

**Published:** 2021-03-25

**Authors:** Hossam El Zayat, Peter Nasr, Hani Sewilam

**Affiliations:** 1 Center for Applied Research on the Environment and Sustainability (CARES), School of Science and Engineering, The American University in Cairo, AUC Avenue, P.O. Box 74, New Cairo, 11835 Egypt; 2 Department of Engineering Hydrology, RWTH Aachen University, Mies-van-der-Rohe Strasse 17, Aachen, 52074 Germany

**Keywords:** Brine management, Desalination, Fertigation, Forward osmosis, FDFO, Reverse osmosis, Volume reduction

## Abstract

A fertilizer drawn forward osmosis (FDFO) process was tested for the concentration of synthetic brine using an industrial-grade fertilizer ammonium sulfate (NH_4_)_2_SO_4_ as the draw solution (DS), NaCl-based synthetic brine as the feed solution (FS), and a commercial forward osmosis (FO) membrane. A bench-scale investigation and a pilot-scale investigation were carried out. By using the highest possible concentration of the DS with a fixed concentration of the FS, the brine generated by reverse osmosis (RO) desalination plants was simulated. The aim of this investigation, performed in batch mode, was to assess the feasibility of using the FDFO process with the tested DS to concentrate the brine by extracting water to dilute the DS. While the main aim of the investigated process was achieving the maximum possible volume reduction of the brine, the resulting DS was further diluted to reduce the nutrients’ concentration in the diluted DS to the acceptable levels producing fertilized water that can be used for fertigation. The investigation showed that the proposed process using the tested fertilizer resulted in an average water flux of 8.01 l/h/m^2^, and a volume reduction of the brine of around 12%.

## Introduction

The production of potable water from both brackish and seawater using membrane desalination technologies, such as RO and nanofiltration (NF), is becoming more common. Nayar et al. (2019) reported that most of the seawater RO plants worldwide operate at an average recovery rate (RR) of 42%, conforming with the range between 35 and 50% reported by McCutcheon et al. (2005). This also matches the range identified by Jones et al. (2019) who suggested typical RRs for other techniques including NF, multi-stage flash distillation (MSF), multiple-effect distillation (MED), and electro-dialysis (ED).

The cost of disposal of seawater desalination brine into the sea is influenced by several factors such as the characteristics and volume of the brine, and the processes of pretreatment and disposal. Generally, the disposal of brine into the sea is estimated to be between 5 and 33% of the total cost of the desalination process, and increases in the case of inland desalination plants (Morillo et al. 2014). In a desalination plant in Ras Shokeir, on the coast of the Red Sea in Egypt, 50,000 m^3^/day, EGP22 million (USD1.4 million), the cost of brine outfall was EGP1.6 million (USD102 thousands), amounting to 7.3% of the total CAPEX of the plant (El-Maraghy 2018). Also, a recent study showed that further extraction of water from desalination brine would result in significant reductions in disposal cost elements such as pipeline length and materials, as well as reductions in pumping energy and jet distance which would allow for the disposal in relatively shallow water bodies (Pistocchi et al. 2020).

However, the discharge of brine into water bodies normally results in a multitude of negative environmental impacts including increased salinity, increased levels of chemicals and metals, aesthetic impacts, and leakage of brine into neighboring aquifers (Missimer and Maliva 2018). Different brine management techniques have been investigated in several studies to reduce the environmental impact of its disposal (Pérez-González et al. 2012; Panagopoulos et al. 2019). Some of the techniques for the management of RO desalination brine are solar evaporation ponds, wind-aided intensified evaporation (WAIV), membrane distillation, forward osmosis, and liquid-liquid extraction.

It was reported by Morillo et al. (2014) that one of the most common approaches to increase the overall RR of an RO plant is through a two-stage RO desalination system in which a secondary RO system is used to further reduce the volume of the brine generated by the primary RO system. Another approach to reduce the environmental impact of brine is through concentration using evaporation ponds, in which the brine is left to evaporate leaving salt to accumulate in the bottom (Morillo et al. 2014; Ahmad and Baddour 2014). According to Mohammadesmaeili et al. (2010), evaporation may also be a practical alternative for brine disposal especially for inland desalination in hot and dry regions. Two major disadvantages of using evaporation ponds are the need for large areas of land and extended time. It was reported by Pérez-González et al. (2012) that the average daily evaporation rate can be as low as 4 l/m^2^. Another disadvantage is the risk associated with potential contamination of the groundwater in the case of possible seepage of the brine into the ground. It was suggested by Sánchez and Matos (2018) that using a series of treatments for salt concentration and the reduction of the volumetric flow rate would facilitate the retrieval of dissolved salts and other compounds through precipitation. They reported that zero liquid discharge (ZLD) could be achieved through a volume minimization process, which should provide the possibility to extract two outputs from one input which is the brine. Supercritical water desalination (SCWD) and SaltWorks crystallizers were also studied by Able and Trembly (2020) for the extraction of water and solid salts from highly concentrated brine to reach ZLD, showing specific energy requirements for both crystallizer and SCWD of 55.3 and 48.7–116.8 kWh/bbl respectively. Membrane distillation (MD) was also investigated by Alrehaili et al. (2020) using a low-temperature water source for RO brine treatment to increase the overall water recovery at a 36,000 m^3^/day, 80–85% recovery RO unit inside a 90,000 m^3^/day wastewater treatment plant. Results showed that MD increased the overall recovery yearly average to 91%, and reduced the volume of the generated brine by 120 million gallons (~ 545,500 m^3^) annually. Earlier studies have shown that the specific energy consumption (SEC) of membrane-based technologies is the lowest compared to other thermal-based technologies for the brine treatment. Typically, SEC of membrane-based technologies is between 0.6 and 19 kWh/m^3^, while for thermal-based technologies, it is between 7.7 and 70 kWh/m^3^ (Panagopoulos et al. 2019).

Since forward osmosis (FO) is driven by the natural osmotic pressure differential between the FS and a draw solution (DS), it offered an energy-efficient approach to extract water from high-salinity solutions using considerably less energy compared to RO, according to McCutcheon et al. (2005) and Su et al. (2012). According to Awad et al. (2019), the six main global suppliers of commercial FO membranes are Fluid Technology Solutions, Modern water, Oasys Water, Porifera, Toyobo, and Trevi Systems. In an FO process, the transfer of water, or water flux, can be calculated using the following equation:

1 $$ {J}_w=A\ \left(\sigma \varDelta \pi -\varDelta P\right) $$

where J_w_ is the water flux, A is the membrane’s water permeability constant, σ is the reflection coefficient, Δπ is the osmotic pressure differential across the two solutions, and ΔP is the externally applied pressure (Nasr and Sewilam 2016b). The osmotic pressure deferential (Δπ) can be calculated as:

2 $$ \varDelta \pi ={\pi}_F-{\pi}_P $$

where π_F_ is the osmotic pressure of the FS and πp is the osmotic pressure of the permeate (Sahebi et al. 2015).

The performance of FO in the extraction of water from brine was tested and compared to other ZLD membrane technologies including RO, high-pressure RO (HPRO), and osmotically assisted RO (OARO) (Panagopoulos et al. 2019). Results showed that FO is more cost-effective and can be applied for water extraction from high-salinity brines (≤ 200 g/l). FO can be used for water extraction for irrigation in a process called fertilizer drawn forward osmosis (FDFO), in which a concentrated fertilizer solution is used as the DS. Since no further processing is required, the diluted DS in an FDFO process could be used directly for fertilized irrigation, or “fertigation.” According to Kafkafi and Tarchitzky (2011), fertigation provides the opportunity to supply the crops directly with the required nutrients through the irrigation water, which can increase the crop yield while maintaining low consumption levels of fertilizers. To optimize the nutrient levels, it was claimed by Phuntsho et al. (2012b) that pre-treatment or post-treatment of feed water, use of blended fertilizer, or hybrid FO system can be used. A study was conducted by Kim et al. (2017) to evaluate and compare the performance of four different reagent-grade fertilizers in two different processes, a standalone FDFO process and a hybrid RO-FDFO process. The results indicated that the hybrid process resulted in more reduced nutrient concentrations compared to the standalone FDFO process producing water that is more suitable for irrigation as a final product.

The efficiency of the FDFO process is highly influenced by both the flux and the reverse permeation of the draw solute from the DS side to the FS side. According to Nasr and Sewilam (2016a) and Nasr and Sewilam (2017), ammonium sulfate exhibited the highest osmotic pressure, low reverse solute permeation, and high rejection of feed solute when compared with urea, ammonium nitrate, and calcium nitrate. While an earlier study showed that the reverse permeation of urea was 29.2 g/l (Phuntsho et al. 2012a), specific reverse solute flux (SRSF) values of less than 0.18 g/l were reported by Nasr and Sewilam (2016a) for ammonium sulfate. In a later study by Wang et al. (2017), ammonium sulfate exhibited the low SRSF compared to other fertilizers: mono-ammonium phosphate (MAP) and potassium phosphate monobasic (KH_2_PO_4_).

As different crops require different (N/P/K) concentrations for their optimal growth, the nutrient levels in the final DS should be compared against the recommended concentrations for the target plant types. For example, it was reported by Phuntsho et al. (2012a) that the recommended (N/P/K) levels for three selected plants (tomato, eggplant, and cucumber) were 200/50/300, 170/60/200, and 200/50/200, respectively. Comparing these figures with the levels of nutrients resulting from the FDFO process, further dilution of the final DS produced by the FDFO process should be required to bring the concentrations closer to the recommended values for those particular plants.

## Materials and methods

This investigation was based on a quantitative method of data collection and analysis through a series of experiments, on both bench-scale and pilot-scale levels, to examine an FDFO process for the volume reduction of synthetic brine as FS and ammonium sulfate as DS.

### Bench-scale investigation

#### Feed solution

The process was tested using a synthetic brine prepared using both reagent-grade and industrial-grade sodium chloride (NaCl) dissolved in deionized (DI) water. The reagent-grade and the industrial-grade NaCl were sourced from Loba Chemie PVT. LTD. and EMISAL Salts Co., Egypt, respectively. The standard specification of the industrial-grade NaCl is shown in Table 1.

**Table 1:** Specifications of industrial-grade NaCl. Based on the datasheet received from EMISAL Salts Co.

Specification	Value
NaCl	Min. 99%
Moisture content	Max. 4.0 %
Impurities soluble in water	Max. 0.8%
Granulometry	< 1 mm (Min. 80% (dried sample))
Appearance	Free-flowing without lumps
Potassium iodate	0.03–0.07 g/l (30–70 ppm)
Water insoluble impurities	< 0.15%
Water soluble impurities	< 1.0%

#### Draw solution

A commonly manufactured ammonium sulfate ((NH_4_)_2_SO_4_) fertilizer was selected as DS for investigation and comparison of results against a reagent-grade fertilizer. The choice of (NH_4_)_2_SO_4_ was due to its price stability and high availability in the local markets (Nasr and Sewilam 2017). Extra pure reagent-grade and granular commercial-grade (NH_4_)_2_SO_4_ were sourced from Loba Chemie PVT. LTD. and Alexandria Fertilizers Co. in Egypt respectively. The specifications of both chemicals are listed in Tables 2 and 3.

**Table 2:** Specifications of reagent-grade ammonium sulfate (LOBA Chemie 2020)

Appearance	Colorless crystals/whitecrystalline powder
Assay (titration)	Min 98.5%
pH (5% solution; 25 °C)	4.5–6.0
Heavy metal (as Pb)	Max 0.002%
Chloride (Cl)	Max 0.003%
Iron (Fe)	Max 0.002%
Sulfated ash	Max 0.05%

**Table 3:** Specifications of industrial-grade ammonium sulfate fertilizer. Based on the datasheet received from Alex Fert Co.

Nitrogen	20.2% min. by weight
Sulfur	24% ± 0.2 by weight
Moisture (H_2_O)	0.5% max. by weight
Sizes < 1.18 mm	0.75% max. by weight
Sizes < 2.00 mm	3.2% max. by weight
Sizes =2.00–4.00mm	90% min. by weight
	Granular, free-flowing, free from harmful substances

#### Membrane

A round commercial FO membrane with a diameter of 40 mm and an effective area of 1.257 × 10^−3^ m^2^, with the specifications listed in Table 4, was used in all bench-scale experiments integrated with a bench-scale crossflow FO system “fluxometer.” The membrane and the fluxometer were both purchased from Porifera Inc, USA.

**Table 4:** Porifera’s FO membrane operating guidelines. Adapted from Porifera Inc (2016) and Nasr (2016)

Item	Specifications
Manufacturer	Porifera Inc.
Model	Roll-to-roll
Pure water permeability coefficient, A (Lm^-2^ h^-1^ bar^-1^)	2.2 ± 0.01
Salt permeability coefficient of active layer, B (m/s)	1.6 × 10.7
Total membrane thickness (μm)	70 ± 10
Structural parameter, S (μm)	215 ± 30
Material of active layer	Polyamide (PA)
Material of support layer	Porous hydrophilic polymer
Water permeation	FO mode: 33 ± 2 LMH PRO mode: 55 ± 3 LMH
Reverse salt flux (RSF)	FO/PRO ,ode: 0.50 ± 0.2 g/L
Membrane parameters	Structural parameter (S value): 215 ± 30 μm
Maximum trans-membrane pressure (TMP)	180 psi
pH operating range	2–11
Maximum chlorine	< 0.1 ppm

#### Methods

**Fig. 1:**
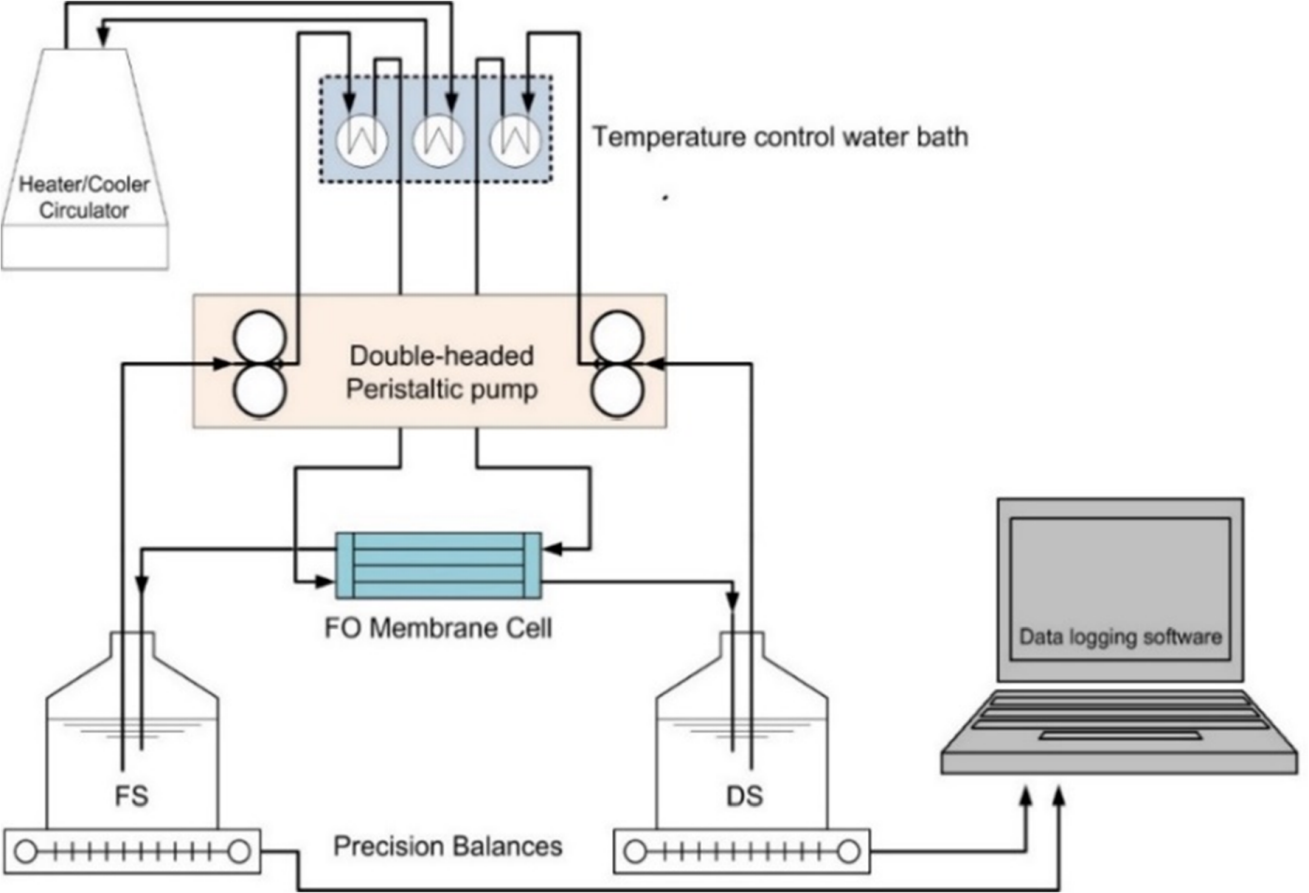
Connection diagram of the FDFO bench-scale experiments

Both the FS and DS were kept flowing in a closed-loop system. As shown in Fig. 1, this system was driven by a double-headed peristaltic pump (Stenner, 170DMP5, USA), from their containers through the pump to the FO cell and back to their containers. To eliminate any effect of possible changes in temperature on the resulting flux, all runs were carried out at a temperature of 25 °C for both solutions using a temperature controller heater/chiller water bath (Polyscience temperature controller, 9106A12E). To calculate the resulting flux, both containers were placed on digital precision balances (Mettler Toledo Precision Balances XS4002S) to give real-time measurements of the change in mass at preset intervals of 120 s. A data-logging software (LabVIEW 2010, 32 bit, Service Pack 1, National Instruments) and an interface management software (Measurement and Automation Explorer MAX, version 15.0.0f0, National Instruments) were used to record the readings of the balances. Average values of all flux calculations for separate mass readings were derived at the end of each experiment.

Mass balance was also monitored throughout the experiments to ensure no leakages in either FS or DS channels. In all experiments, carried out in batch mode, the initial volumes of both FS and DS were set at 200 ml each. As the FO process continued, the DS was being continuously diluted and the concentration of FS was being increased. The resulting decline in the effective osmotic deferential caused a gradual decrease in the rate at which freshwater was transferred from the FS side to the DS side. For around 60 min, while observing the change in mass, water flux was calculated and plotted against time. A sample was taken from each solution before and after each run to record both conductivity and total dissolved solids (TDS) values and to measure the concentration of the nutrients in the final DS.

To measure conductivity and TDS values throughout the investigation, Orion Star A325 pH/Conductivity Portable Meter and Orion DuraProbe™ Conductivity Cells by Thermo Fisher Scientific Inc. were used. The stirrer (Stuart Hotplate stirrer – UC 152) was used to prepare the solutions. The speed was increased gradually from the beginning to the end of each mixing procedure from 100 to 300 rpm to allow for a slow dissolution of the chemicals. The runtime of the last experiment was 36 h instead of only 1 h for earlier runs. To secure an adequate amount of FS to accommodate the expected transfer of water, the initial volume of the FS in the last run was 2 l while that of the DS was 200 ml. The water flux J_w_ was calculated as follows:

3 $$ {J}_W=\frac{\Delta  V}{A\ast T} $$

Where

∆*V*: *Change in solution volumes*
*A*: *Area of the FO membrane in m2*
*T*: *Time interval in hours*

A baseline experiment was carried out to test the membrane’s basic operation and set baseline values for the generated water flux using deionized (DI) water as FS with reagent-grade NaCl as DS in one run and reagent-grade (NH_4_)_2_SO_4_ as DS in a second run. Then, three experiments were conducted as explained below and summarized in Table 5.

**Table 5:** Experiments of the bench-scale investigation

Experiment	FS	DS
Baseline experiment	DI	58.44 g/l (1 mol) reagent-grade NaCl, 132.14 g/l (1 mol) reagent-grade (NH_4_)_2_ SO_4_, and 740 g/l industrial-grade (NH_4_)_2_ SO_4_
Experiment (I)	Reagent-grade NaCl @ 5, 35 and 65 g/l (5,000, 35,000, and 65,000 ppm)	132.14 g/l (1 mol) and 396.42 g/l (3 mol) reagent-grade (NH_4_)_2_ SO_4_
Experiment (II)	Reagent-grade NaCl @ 65 g/l (65,000 ppm)	740 g/l industrial-grade (NH_4_)_2_ SO_4_
Experiment (III)	Industrial-grade NaCl @ 65 g/l (65,000 ppm)	740 g/l industrial-grade (NH_4_)_2_ SO_4_

First, reagent-grade NaCl and (NH_4_)_2_SO_4_ were used as FS and DS respectively. The reagent-grade NaCl was used at three different concentrations (5, 35, and 65 g/l) and the reagent-grade (NH_4_)_2_SO_4_ was used as DS at three concentrations (132.14, 264.28, and 369.42 g/l) (equivalent to 1, 2.4, and 3 mol respectively). Both FS and DS were prepared using DI water throughout this set of experiments. A flushing procedure was used to remove any remaining chemicals in the FS and DS channels and the membrane. The fluxometer and the membrane were flushed using DI twice after each run and before the start of the following run. This process was repeated until the TDS reading of the water in both channels was less than 1.0 ppm before starting the following run.

Then, in the following set of 3 experiments, industrial-grade (NH_4_)_2_SO_4_ was used as DS, while the reagent-grade NaCl continued as FS at a concentration of 65 g/l to simulate RO brine. The concentration of the DS was set at 740 g/l, the maximum possible concentration of the industrial-grade (NH4)_2_SO_4_ without heating. This concentration resulted from a parallel experiment where the mass of the solute was increased gradually until no further dissolution was possible. In the final stage, reagent-grade NaCl was replaced by industrial-grade NaCl as FS, while industrial-grade (NH_4_)_2_SO_4_ was maintained as the DS. The concentration of FS was set at 65 g/l to simulate RO brine while that of DS was set at 740 g/l. For all experiments, the change in the masses of FS and DS, and the resulting flux were recorded.

### Pilot-scale investigation

The pilot-scale investigation was conducted by scaling up the findings of the bench-scale stage using a pilot-scale FO arrangement. While for the baseline experiments DI water was used to eliminate the possible effects of any suspected presence of impurities while establishing the baseline values, the final experiment was conducted using tap water instead of DI water to simulate a scenario closer to reality.

#### Feed and draw solutions

All experiments were carried out in batch mode using industrial-grade NaCl for the preparation of synthetic brine as FS. Table 6 shows a summarized list of the experiments carried out as part of this investigation. The industrial-grade NaCl was sourced from EMISAL Salts Co., Egypt, with the standard specifications listed in Table 1. Industrial-grade (NH_4_)_2_SO_4_ was used as DS at the maximum concentration. On the other hand, DI was used as FS in the baseline experiment then it was replaced with industrial-grade NaCl at a concentration of 65 g/l dissolved in DI. The industrial-grade fertilizer (NH_4_)_2_SO_4_ was used in both pilot- and bench-scale investigations.

**Table 6:** Experiments of the pilot-scale investigation

Pilot-scale stage	FS	DS
Baseline experiment	DI	Industrial-grade (NH_4_)_2_ SO_4_ @ 740 g/l
Main experiment	Industrial-grade NaCl @ 65 g/l (65,000 ppm)	Industrial-grade (NH_4_)_2_ SO_4_ @ 740 g/l

#### Membrane

**Fig. 2:**
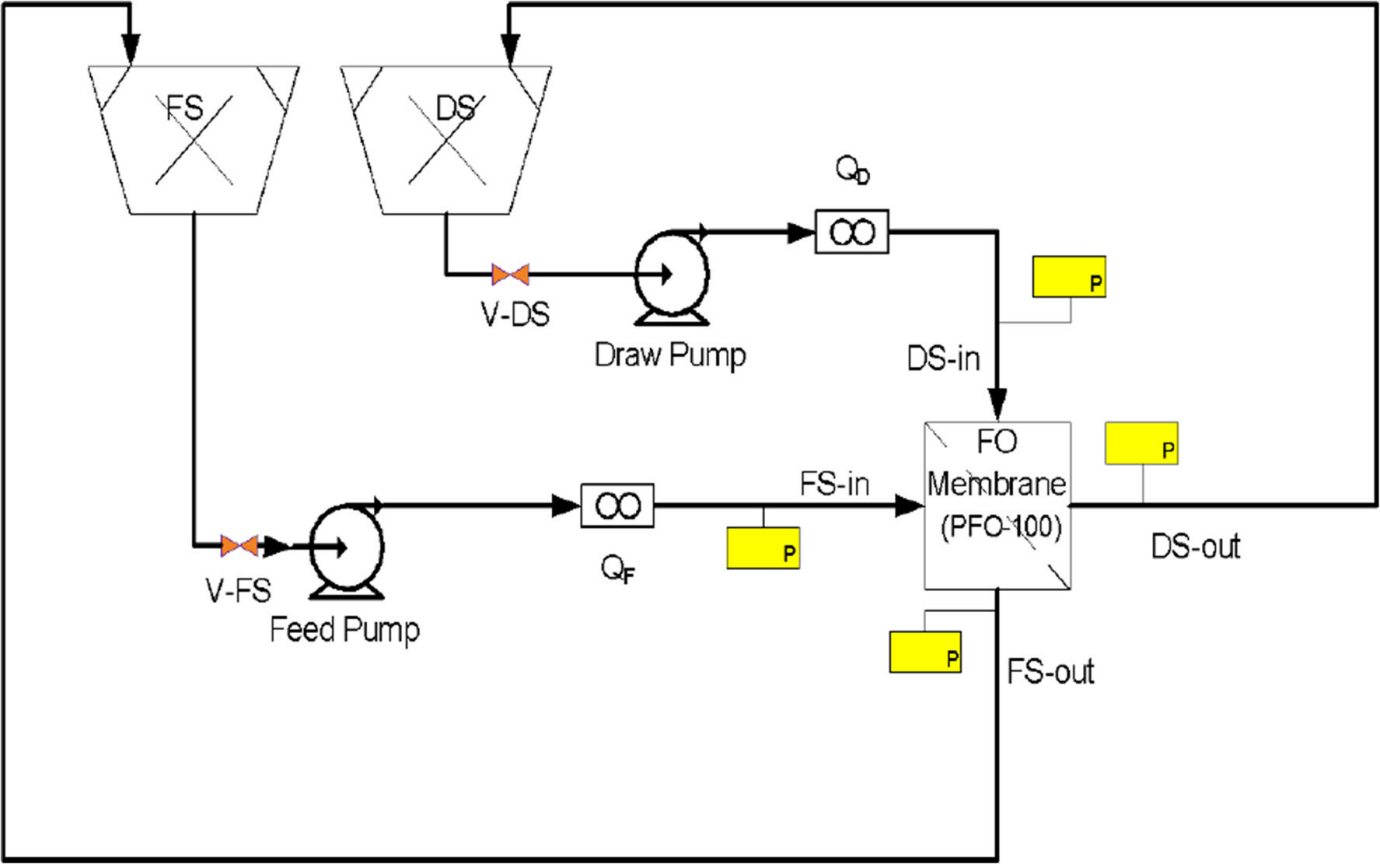
Connection diagram of the FDFO pilot-scale experiments

The pilot-scale experiments used the FO arrangement shown in Fig. 2 at the Water-Energy-Food Nexus Lab at The American University in Cairo. The FO arrangement includes a commercial 7 m^2^ FO membrane module supplied by Porifera Inc, USA, with the specifications listed in Table 4. The modular design of the membrane allows for the future addition of more membrane modules to increase the effective area in multiples of 7 m^2^. Each module has input connections on one side and the output connections on the other, and include four ports on each side: two for FS and two for DS. Two of the four ports on each side were connected to pressure gauges to continuously monitor the pressure of both solutions and to ensure they do not exceed the maximum allowable pressure limit. Two digital flow meters were installed on the input side of both FS and DS. Deionized water was used for the flushing of the membrane between runs and was circulated for the removal of foulants.

#### Methods

The arrangement was powered by two 0.55-kW circulation pumps where each pump was connected in line with the feed or the draw tanks. The pumps were selected so that the pressure on the input ports of the membrane module does not exceed 1 bar and are controlled by two pressure switches. A control board was installed as part of the arrangement providing the option of operating the system manually or through a Programmable Logic Control (PLC) system. To be fully in control of the process, only the manual mode option was used throughout the investigation. A platform scale was used to weigh and record the changes in the mass of the DS throughout each run. The scale and the data logging software were supplied by Western Mechatronics. The data logger was set to display and record the weight of the DS every 1 min from the start until the end of each experiment and export data to an Excel file for analysis. The mixing of both the DS and FS was performed using the Heidolph Hei-TORQUE Precision 400 overhead stirrer, and increasing the mixing speed gradually from 500 to 1200 rpm. The 900-W, 4100 l/h Einhell GE-GP 9041 E pump was used in the pilot-scale experiments for two functions. The first function was to prepare of the synthetic brine by mixing the industrial-grade NaCl in water. The second function was to transfer the prepared FS from the mixing container to the FS container before the start of each run. Two 200-l tanks were used for DS and FS. Each tank was connected to a dedicated circulation pump which provided the driving force to move each solution from the base of the container through the membrane to the top of the container. Before the start of each run, the system was flushed using DI until the TDS reading of both channels was below 100 ppm (0.1 g/l). The initial volume of both solutions was recorded and the change in the volume of the DS was observed by continuously monitoring and recording the change in its mass using the platform scale. The recorded change in the DS volume was used to calculate the flux as the rate of the transfer of freshwater from the FS side to the DS side per unit area per hour. The effectiveness of the process on the volume of the FS was tackled by quantifying the change in the volume and TDS concentration of the FS before and after the process compared to their initial values.

A baseline experiment was conducted using DI as FS and industrial-grade (NH_4_)_2_SO_4_ as DS at the maximum concentration. The purpose of this experiment was to establish the baseline value of the resulting flux to be generated using the investigated fertilizer as DS. The resulting flux was expected to be the maximum possible flux. The initial volume of the DS was 10 l while the initial volume of the FS was 200 l. Two experiments were carried out in which industrial-grade NaCl and (NH_4_)_2_SO_4_ were used as FS and DS respectively. The concentration of FS was set at 65 g/l while DS was prepared at 740 g/l, the maximum concentration of the (NH_4_)_2_SO_4_. One experiment used both NaCl and (NH_4_)_2_SO_4_ dissolved in DI. The other experiment used tap water only. Tap water was de-chlorinated before adding and dissolving both solutes as per the membrane supplier instructions. A parallel experiment investigated the effect of the de-chlorination method on the TDS and pH readings using three different techniques: sodium thiosulphate (Na_2_S_2_O_3_), sodium metabisulfite (Na_2_S_2_O_5_), and de-chlorination by evaporation. Based on the results, and to avoid the need for a pre-treatment stage only for de-chlorination, the evaporation method was selected after it was tested and proven successful.

## Results and discussion

### Bench-scale investigation

#### Change in flux and concentration

**Fig. 3:**
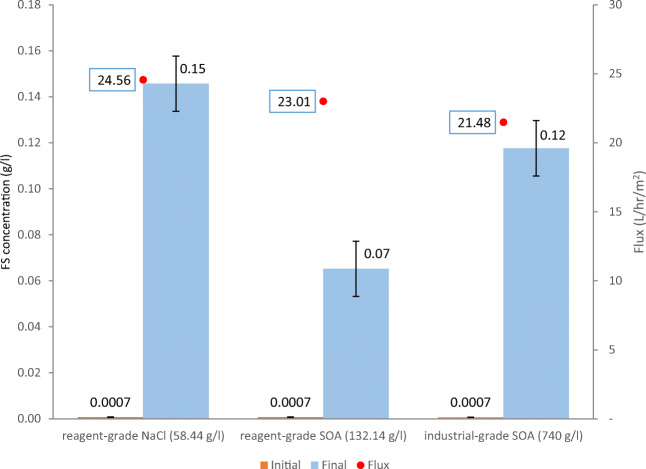
Water flux and FS concentration before and after the baseline experiment with DI used as FS and different DS

The first baseline experiment was carried out to identify the maximum concentration of the industrial-grade (NH_4_)_2_SO_4_ in DI which was expected to yield the highest water flux. The experiment results show that the tested industrial-grade (NH_4_)_2_SO_4_ can reach a maximum concentration of 740 g/l when dissolved in DI at ambient temperature, without heating. The following experiment involved using DI as FS in three successive runs while the DS was changed from 58.44 g/l (1 mol) reagent-grade NaCl, to 132.14 g/l (1 mol) reagent-grade (NH_4_)_2_SO_4_ to industrial-grade (NH_4_)_2_SO_4_ at the maximum concentration (i.e., 740 g/l). The conductivity, TDS readings, and the change in the masses of FS were recorded for each experiment. As shown in Fig. 3, water flux was derived for each experiment and plotted from the recorded values of the change in mass.

Using reagent-grade NaCl and (NH_4_)_2_SO_4_ as DS at a concentration of 1 mol, the water fluxes were 24.56 and 23.01 l/h/m^2^ respectively while the volumes of filtration were 31.67 ml (15.5%) and 31.05 ml (15.8%) respectively. Using industrial-grade (NH_4_)_2_SO_4_ as DS at the maximum concentration (i.e., 740 g/l), flux was only 21.48 l/h/m^2^. The concentration of 740 g/l of reagent-grade (NH_4_)_2_SO_4_ is around 5.4 mol, more than five times the tested concentration of the reagent-grade (NH_4_)_2_SO_4_. Accordingly, it was assumed that the impurities in the industrial-grade (NH_4_)_2_SO_4_ caused the osmotic pressure to drop significantly causing the draw action to drop as well.

**Fig. 4:**
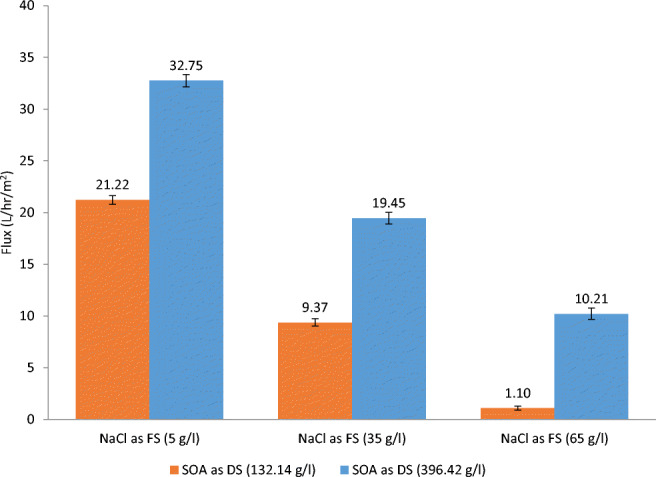
Water flux with two concentrations of reagent-grade SOA as DS and reagent-grade NaCl as FS at three different concentrations

Then, DI was replaced with a reagent-grade NaCl at three different concentrations (5, 35, and 65 g/l) as FS and was tested with reagent-grade (NH_4_)_2_SO_4_ as DS at two concentrations (132.14 and 396.42 g/l (equal to 1 and 3 mol respectively)). As shown in Fig. 4, the resulting six different concentration combinations yielded water flux levels ranging from a low of 1.10 l/h/m^2^ to a high of 32.75 l/h/m^2^.

**Fig. 5:**
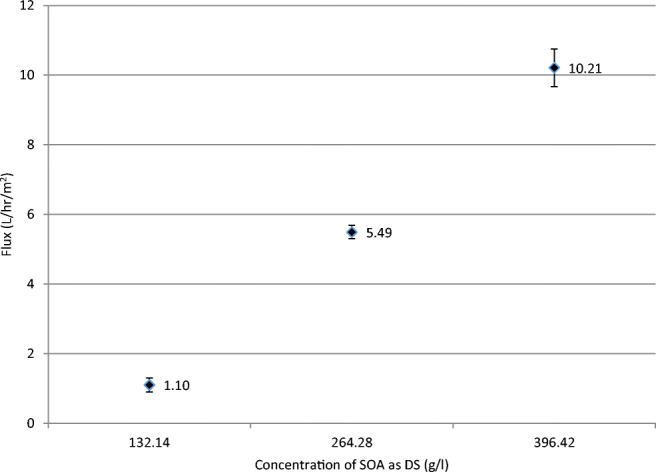
Water flux using reagent-grade NaCl as FS at 65 g/l at three concentrations of reagent-grade SOA as DS

Since the main focus of this research is the volume reduction of RO brine, further investigation was performed on the third case. The concentration of the reagent-grade NaCl as FS was set at 65 g/l to simulate seawater brine and the concentration of the reagent-grade (NH_4_)_2_ SO_4_ as DS was changed from 132.14 to 396.42 g/l, equivalent to a change from 1 to 3 mol. The resulting flux changed, as shown in Fig. 5, from 1.10 to 10.21 l/h/m^2^.

**Fig. 6:**
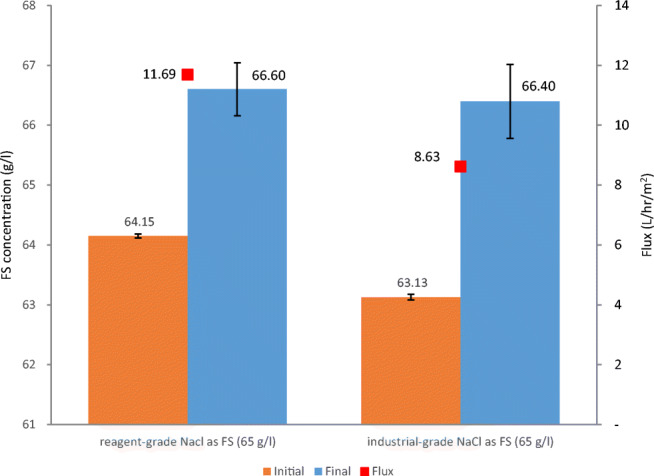
Water flux and FS concentration before and after the final bench-scale experiment with industrial-grade SOA @ 740 g/l as DS

A further investigation was conducted to study the effect of changing the solute used to prepare the FS from reagent to industrial-grade NaCl on the generated water flux and the achieved concentration of FS. As shown in Fig. 6, the reagent-grade NaCl at a concentration of 65 g/l was used as FS with industrial-grade (NH_4_)_2_SO_4_ as DS at a concentration of 740 g/l. The resulting water flux was 11.69 l/h/m^2^ compared to only 8.63 l/h/m^2^ when industrial-grade NaCl was used at the same concentration. On the volume reduction level, the change in TDS measurements for both FS and DS showed that industrial-grade NaCl as FS resulted in a concentration of the FS of (5.2%) compared to only (3.8%) respectively when reagent-grade NaCl was used as FS.

**Fig. 7:**
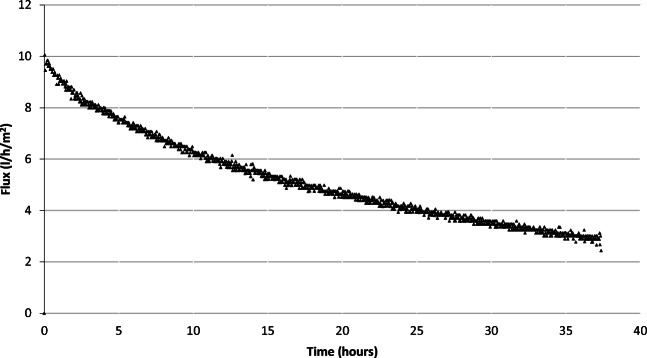
Flux vs time for the FO process using industrial-grade NaCl at a concentration of 65 g/l as FS and industrial-grade SOA at a concentration of 740 g/l as DS

An additional experiment was carried out for an extended period of time to identify the long-term change in water flux and concentrations. The initial volumes of the FS and DS were 2 l and 200 l, respectively. The experiment was kept running for 36 h instead of 1 h. The results showed that the flux slightly decreased from 8.63 l/h/m^2^ in a 1-h run to 8.09 l/h/m^2^ in the extended run. The change in flux value against time is depicted in Fig. 7.

**Fig. 8:**
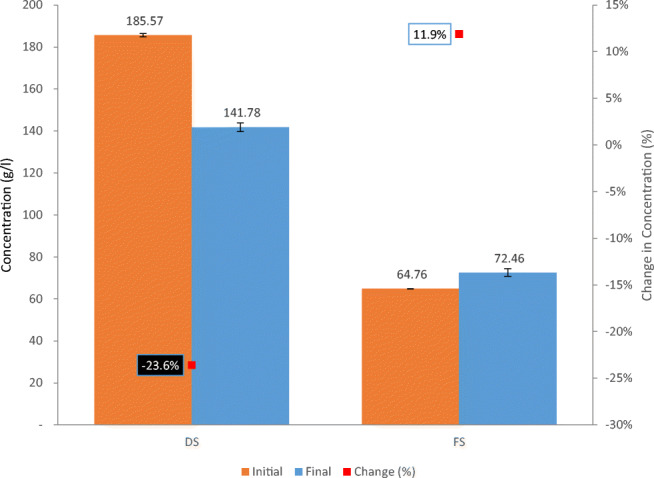
Absolute and % change of concentration of both FS and DS before and after the extended run using industrial-grade NaCl at a concentration of 65 g/l as FS and industrial-grade SOA at a concentration of 740 g/l as DS

A comparison between the TDS measurements before and after the extended run for both solutions is shown in Fig. 8. Throughout the 36-h runtime, flux was continuously decreasing as a result of approaching osmotic equilibrium between the two solutions. By the end of the run, no significant flux was observable while 245 g of freshwater (12.3% of the initial volume of the FS) was transferred from the feed side into the draw side. To confirm the percentage of volume reduction, an approximately equal percentage could be derived using another approach. By analyzing the change in TDS measurements of the FS before and after the run, the concentration of the FS was increased by 11.9%, from 64.7 to 72.4 g/l.

### Pilot-scale investigation

#### Change in flux

**Fig. 9:**
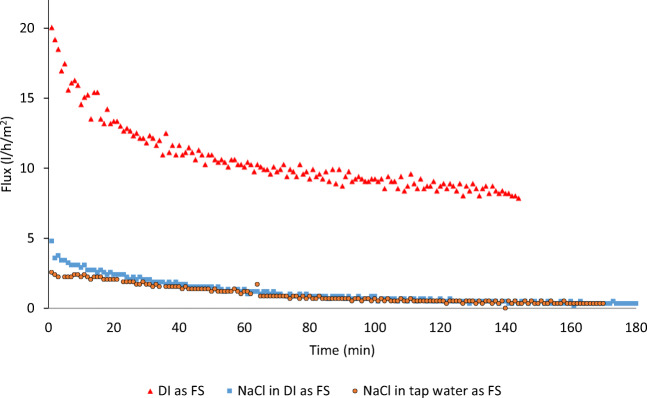
Flux vs time with industrial-grade (NH_4_)2SO_4_ (740 g/l) and DI 65 g/l DI-based NaCl solution, and 65 g/l tap water-based NaCl solution as FS

**Fig. 10:**
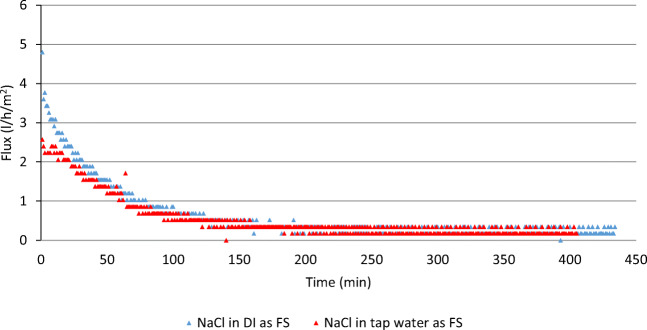
Flux vs time with industrial-grade (NH_4_)2SO_4_ as DS at a concentration of 740 g/l and NaCl as FS at a concentration of 65 g/l

The investigation started with an initial concentration of the DS set at 740 g/l, the maximum possible concentration of the investigated fertilizer in water. The experiment was run for 180 min while recording the change in the mass of the DS with resulting flux of 14.39 l/h/m^2^. In the following two experiments, the FS was replaced with industrial-grade NaCl solution at a concentration of 65 g/l which was dissolved in DI in one experiment and tap water in another experiment. The process was repeated as explained in the baseline experiment and the resulting flux values were 2.40 l/h/m^2^ (with DI-based FS) and 2.02 l/h/m^2^ (with tap water-based FS) as shown in Fig. 9. Then, two extended runs using industrial-grade (NH_4_)_2_SO_4_ as DS (740 g/l) and industrial-grade NaCl at a concentration of 65 g/l as FS dissolved in DI and tap water investigated the long-term results of flux and the change in the TDS of both solutions. When both runs extended to 7 h, average flux dropped to 1.52 l/h/m^2^ (with DI-based FS) and 1.33 l/h/m^2^ (with tap water-based FS) as shown in Fig. 10. The slight decrease in the resulting flux values with the use of the tap water-based FS was expected due to the higher TDS of the tap water compared to the DI water. In addition, possible impurities caused the osmotic pressure of the FS to slightly increase thus decreasing the overall driving osmotic pressure difference and lowering the resulting flux.

#### Change in concentration/volume due to permeation

Table 7 shows the initial and final TDS readings of both FS and DS and the corresponding change in the concentration of each solution. The process resulted in an average concentration of the FS of around 12.7%. The changes in the TDS readings of both the FS and DS were compared against the recorded changes in the volume of both solutions. Table 8 shows the initial and final volumes of both FS and DS and the corresponding change in the volume of both solutions. The change showed that the tested process resulted in a reduction in the FS volume of 12.4%, a result that is conforming to the recorded change in TDS readings.

**Table 7:** Change in the concentrations of FS and DS (g/l) and flux (l/h/m^2^) of the pilot-scale investigation

Description	DS concentration (g/l)		FS concentration (g/l)		Flux (l/h/m^2^)	Change in concentration	
	Initial	Final	Initial	Final		DS	FS
Industrial-grade (NH_4_)_2_ SO_4_ (740 g/l) as DS and industrial-grade NaCl (65 g/l) dissolved in DI as FS	186.59	85.19	63.29	71.91	1.52	− 54.3%	13.6%
Industrial-grade (NH_4_)_2_ SO_4_ (740 g/l) as DS and industrial-grade NaCl (65 g/l) dissolved in tap water as FS	187.48	89.48	65.72	73.41	1.32	− 52.3%	11.7%
Average						− 35.3%	12.7%

**Table 8:** Recorded changes in FS and DS volumes

Parameter	DS	FS
Initial volume (liter)	10.00	200
Final volume (liter)	34.80	175.20
Extracted fresh water (liter)	− 24.80	24.80
Volume reduction of FS		12.4%

### Projected productivity of the process

**Fig. 11:**
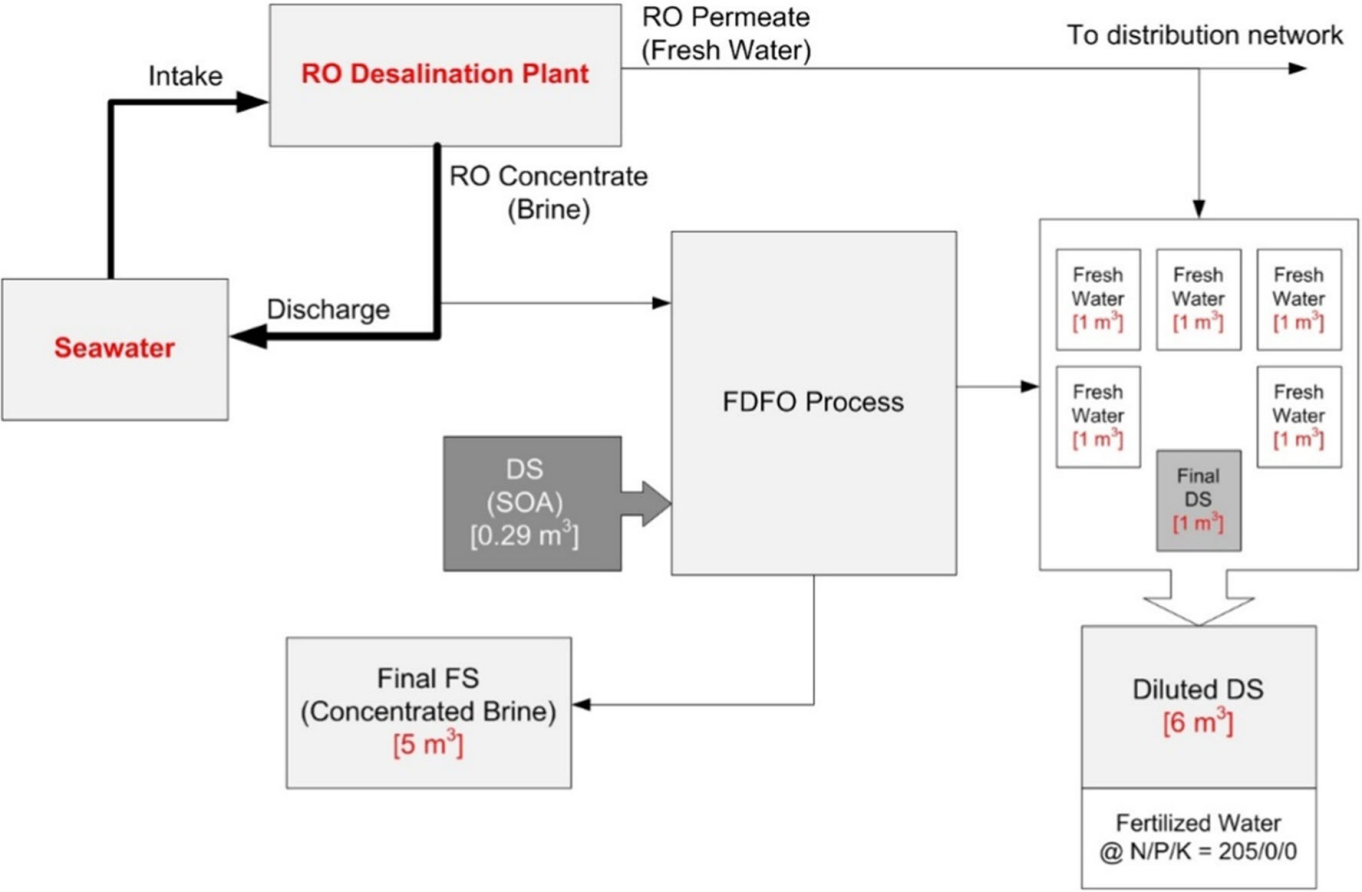
Mass balance of the proposed process

Based on the results obtained from the change in the concentrations of both FS and DS in the final experiment of the pilot-scale stage, a case was derived to calculate the required DS quantity which would produce 1 m^3^ of diluted DS. The overall proposed systems layout was developed using the quantities illustrated in Fig. 11. The calculations showed that to produce 1 m^3^ of diluted DS, 0.29 m^3^ of the concentrated initial DS is required to be used with 5.75 m^3^ of RO brine at a concentration of 65 g/l. This should result in an overall volume reduction of the feed brine of 12.4% to reach 5 m^3^ at a concentration of 73 g/l. The resulting concentrated brine can be directed toward solar evaporation ponds or used in aquaculture farming processes. Post-treatment of the resulting concentrated brine might be required to precipitate any traces of sulfates before using it in any fish farming processes. Based on the results of the analysis of a sample of the final DS conducted at the Agricultural Research Center (ARC) in Cairo, the concentration of nitrogen (N) was found to be 1200 mg/l. Accordingly, in this particular scenario, the final DS will need to be further diluted using 5 m^3^ of freshwater (RO permeate) to bring the final concentration of nitrogen down to 205 mg/l, a concentration that meets the recommended N/P/K nutrients’ concentrations for the plants (Phuntsho et al. 2012a).

### Economic assessment

Different cost elements were used to assess the cost of the proposed FDFO process. Table 9 lists different cost elements from the local market and using an exchange rate of 15.7 EGP per US dollar. The cost of the ammonium sulfate was identified from the local market, while the cost of desalinated water (RO permeate) and the cost of electricity were according to market averages and were confirmed through an interview with an industry expert (Seddik 2018).

**Table 9:** Different parameters used in the calculation of the cost elements of the proposed FDFO process

Parameter	Unit	Value
Cost of fertilizer	USD/kg	0.23
Cost of freshwater (RO permeate)	USD/m^3^	0.61
Cost of electricity	USD/kWh	0.10
Rated power of one pump	kW	0.55
Weight of 10 l initial DS	kg	12.42
DS initial weight	kg	12.42
DS final weight	kg	29.92
Time consumed	h:min	7:25
Initial concentration of SOA	kg/l	0.74

A case was derived based on the results obtained from a pilot-scale experiment where 10 l of industrial-grade fertilizer was used as initial DS and 200 l of synthetic brine was used as initial FS. The projected amounts of freshwater and solid fertilizer in this case were calculated so that the resulting final DS was equal to 1 m^3^ (Table 10). Also, different cost and saving elements were calculated and split into CAPEX and OPEX as shown in Table 11. The results showed that the proposed method contributed to the reduction of the RO plant’s CAPEX by USD 20.29 per m^3^ of plant’s capacity. This is a direct result of reducing the cost of the required brine outfall due to the reduced volume of the brine. On the OPEX side, the proposed method is expected to result in total OPEX of USD 36.43 per m^3^ and to reduce the cost of brine disposal by USD 0.08 for every cubic meter of the final diluted DS. More savings on other cost elements such as brine pipeline material and pumping energy are expected to further enhance the economic viability of the proposed process. Also, the revenues generated from the commercialization of the produced fertilized water will represent a significant improvement to the overall economic viability of the process.

**Table 10:** Parameters used for the derived case based on the results of the pilot-scale experiment

	Experiment		Derived case to reach 1 m^3^ of final DS		
	DS	FS	Freshwater	DS	FS
Initial volume (l)	10.00	200	208.99	287.36	5747.13
Final volume (l)	34.80	224.80		1000.00	5034.48
Extracted freshwater (l)	24.80	24.80		712.64	712.64
Volume reduction of FS (%)		12.4			12.4%
Fertilizer mass (kg)				154.65	
Water extraction capacity of fertilizer (l/kg)				4.61

**Table 11:** Itemized costs and savings involved in an FDFO process

Costs		Source
CAPEX		
Cost of FO membrane (incl. shipping) (USD)	5900	Porifera Inc (2021)
OPEX (/m^3^)		
Cost of water (USD)	0.13	
Cost of fertilizer (USD)	35.46	
Cost of energy (circulation pumps) (USD)	0.83	
Total OPEX (USD/m^3^)	36.42	
Savings		
CAPEX		
Average share of brine outfall in total RO plant CAPEX	7.30%	N. El-Maraghy (2018)
Average cost of RO plant CAPEX (USD/m^3^/day)	2242	Judd (2017)*
Average cost of brine outfall (USD/m^3^)	163.67	
Savings in CAPEX (USD/m^3^)	20.29	
OPEX (/m^3^)		
Average cost of brine disposal in total desalination cost (%) [min 5% & max 33%]	19%	Pérez-González et al. (2012)
Average cost of brine disposal (USD/m^3^)	0.11	
Average savings on the disposal of the reduced brine volume (USD)	0.08	

*Based on the average CAPEX of 5 RO plants in Ashkelon (330 MLD), Tuas (110 MLD), Perth (144 MLD), Sydney (250 MLD), and Sorek (624 MLD)

## Conclusions

The bench-scale investigation was successful in identifying the maximum possible concentration of the tested industrial-grade (NH_4_)_2_SO_4_ in water, without the introduction of any external heating, which was found to be 740 g/l. To achieve the most powerful draw action using this particular fertilizer in an FDFO process, the DS was prepared at the identified maximum concentration. Testing the process using several bench-scale investigations was useful on different levels. One outcome of the bench-scale investigations was that when the fertilizer was used as DS with synthetic brine prepared using reagent-grade NaCl as FS, the generated flux was 11.69 l/h/m^2^ compared to 8.63 l/h/m^2^ using an industrial-grade NaCl as FS. It can be assumed that the impurities in the industrial-grade NaCl increased its osmotic pressure which resulted in decreasing the osmotic pressure differential between the FS and DS sides and, accordingly, reducing water flux. Future research may be conducted to verify this assumption by fully analyzing two samples of both the reagent-grade and the industrial-grade NaCl to identify the exact chemical composition of both salts which can help explain the observed decrease in flux.

Based on the extended 36-h run, another notable observation is that the FDFO process using industrial-grade NaCl at a concentration of 65 g/l and (NH_4_)_2_SO_4_ at a concentration of 740 g/l as FS and DS respectively resulted in an increase in the concentration of the synthetic brine of around 12%. This observation translates into a volume reduction of the brine of the same percentage. By quantifying the percentage of freshwater which has been transferred from the FS to the DS, to the initial volume of the FS, the same percentage was confirmed. This reduction in volume translates into improved economics of disposal of the concentrated brine using evaporation ponds.

The pilot-scale investigation showed that the FDFO process using industrial-grade (NH_4_)_2_SO_4_ at a concentration of 740 g/l as DS for the volume reduction of synthetic brine as FS resulted in an increase in the concentration of the FS ranging between 12.4 and 12.7%. The observed change in both TDS readings and volume of the FS conformed with the results of the bench-scale stage showing a 12% volume reduction of the FS. The achieved volume reduction of 12.7% using the proposed process, an RR of 12.7%, can be considered comparable in terms of the overall economic viability of the process, although it is lower than the reported 19% (Jones et al. 2019). The reported RR of 19% was in fact linked to an RO process that normally requires a significantly high amount of energy unlike the case with the proposed FDFO process. An advantage of the proposed FDFO process over the reported RO is that the former produces concentrated fertilized water which can be commercialized to provide an additional revenue stream enhancing the overall economic viability of this process. Compared to recent ZLD techniques such as crystallizer and SCWD, the proposed process showed lower levels of energy consumption per cubic meter. The energy consumed in the FDFO process, mainly by the circulation pumps, was 8.16 kWh for the extraction of 24.8 l. For the derived case, the projected cost of energy is 33.53 USD/m^3^, lower than the reported energy costs of 47.3 and 41.66–99.8 USD/m^3^ associated with crystallizer and SCWD respectively (Able and Trembly 2020).

Although a higher concentration rate of brine might be achievable if a higher volume of concentrated draw is used, the scope of this study was meant to investigate the results given the conditions pointed out in the “Materials and methods” section. A further study involving larger volumes of DS is recommended to investigate possibly reaching higher concentrations of brine.

It can be concluded that the overall outcomes of both investigations showed consistent results in terms of the volume reduction of desalination brine used as FS. This volume reduction is expected to yield a multitude of positive results, including sustainable management of brine as well as an increased possibility of the recovery of salts and value-added chemicals, which can be recovered in later stages using different techniques such as evaporation and crystallization.

## Data Availability

The datasets used and/or analyzed during the current study are available from the corresponding author on reasonable request.
